# Successful conversion surgery for unresectable gastric cancer with giant para-aortic lymph node metastasis after downsizing chemotherapy with S-1 and oxaliplatin: a case report

**DOI:** 10.1186/s40792-018-0494-4

**Published:** 2018-08-07

**Authors:** Akiko Serizawa, Kiyoaki Taniguchi, Takuji Yamada, Kunihiko Amano, Sho Kotake, Shunichi Ito, Masakazu Yamamoto

**Affiliations:** Department of Surgery, Institute of Gastroenterology, Tokyo Women’s Medical University, 8-1, Kawada-cho, Shinjuku-ku, Tokyo, 162-8666 Japan

**Keywords:** Remnant gastric cancer, Para-aortic lymph node metastasis, Pathological complete response

## Abstract

**Background:**

Although patients with stage IV gastric cancer who respond well to systemic chemotherapy can be treated with gastrectomy, the prognosis of patients with unresectable gastric cancer with para-aortic lymph node metastasis is poor. We herein report a case of remnant gastric cancer with para-aortic lymph node metastasis that was treated with potentially curative conversion surgery after showing a complete response to chemotherapy with S-1 and oxaliplatin (SOX).

**Case presentation:**

An 81-year-old man was diagnosed with type 3 remnant gastric cancer with giant para-aortic lymph node metastasis, and he received SOX chemotherapy. After three courses of SOX chemotherapy, the primary tumor and para-aortic lymph node metastases markedly reduced in size, indicating a partial response. Because conversion surgery was possible, the patient underwent total remnant gastrectomy with D2 and para-aortic lymph node dissection. Histological examination revealed no residual cancer cells in the resected stomach and lymph nodes. The patient was diagnosed with a complete pathological response and was discharged on postoperative day 24. Currently, 1 year after surgery, the patient is alive and has not shown any tumor recurrence.

**Conclusion:**

To the best of our knowledge, this is the first case of advanced remnant gastric cancer with giant para-aortic lymph node metastasis that showed a pathological complete response and favorable outcome after SOX chemotherapy.

## Background

Remnant gastric cancer (RGC) with para-aortic lymph node (PAN) metastasis is classified as stage IV cancer by using the 7th Union for International Cancer Control (7th UICC) guidelines. Furthermore, surgery is not indicated for stage IV gastric cancer according to the Japanese gastric cancer treatment guidelines [1], and the prognosis is extremely poor [2]. Moreover, no evidence-based preoperative chemotherapy regimens are available for treatment. As the first-line treatment for advanced gastric cancer, the S-1 and oxaliplatin (SOX) regimen was almost as effective as the S-1 and cisplatin (SP) regimen [3], but with a more favorable safety profile.

Herein, to the best of our knowledge, we report the first case of advanced RGC with giant PAN metastasis that showed a complete pathological response and favorable outcome after SOX chemotherapy.

## Case presentation

**Fig. 1:**
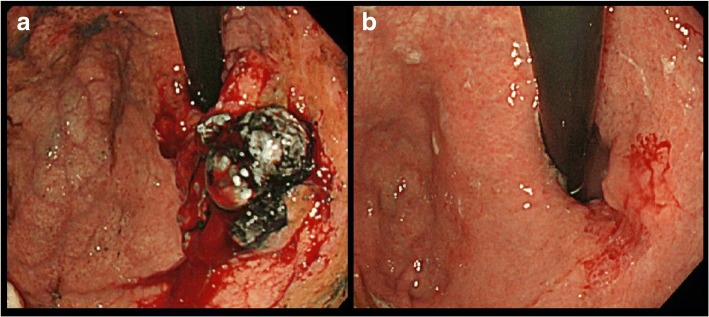
Endoscopy findings. **a** Endoscopy before chemotherapy revealed Borrmann type 3 cancer in the lesser curvature of the stomach. **b** Endoscopy after chemotherapy showed a scar-like flat lesion in the lesser curvature of the remnant stomach

**Fig. 2:**
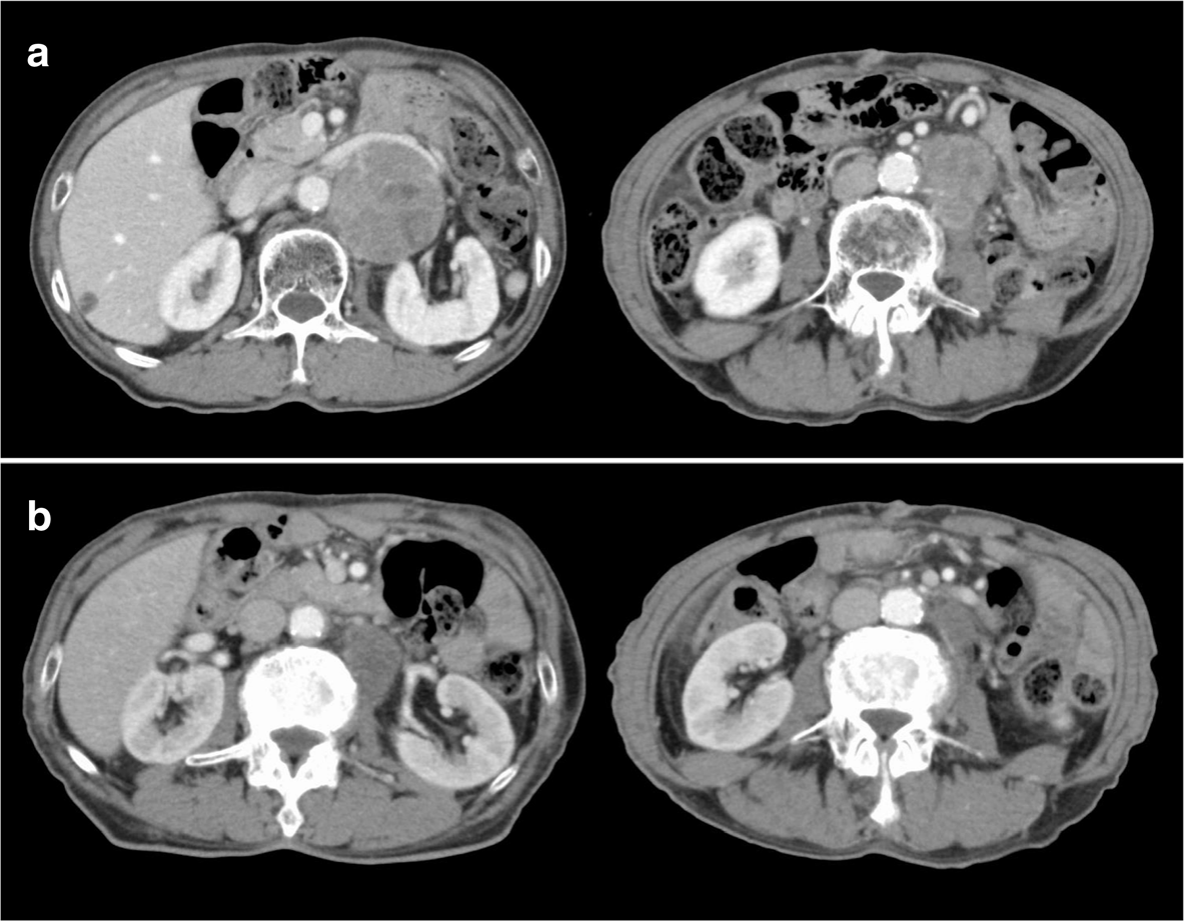
Computed tomography (CT) findings. **a** Abdominal contrast-enhanced CT before chemotherapy showed that the para-aortic lymph nodes were swollen (No. 16a2 70 mm, No. 16b1 30 mm respectively). CT after chemotherapy revealed that they were reduced in size (No. 16a2 (**b**))

**Fig. 3:**
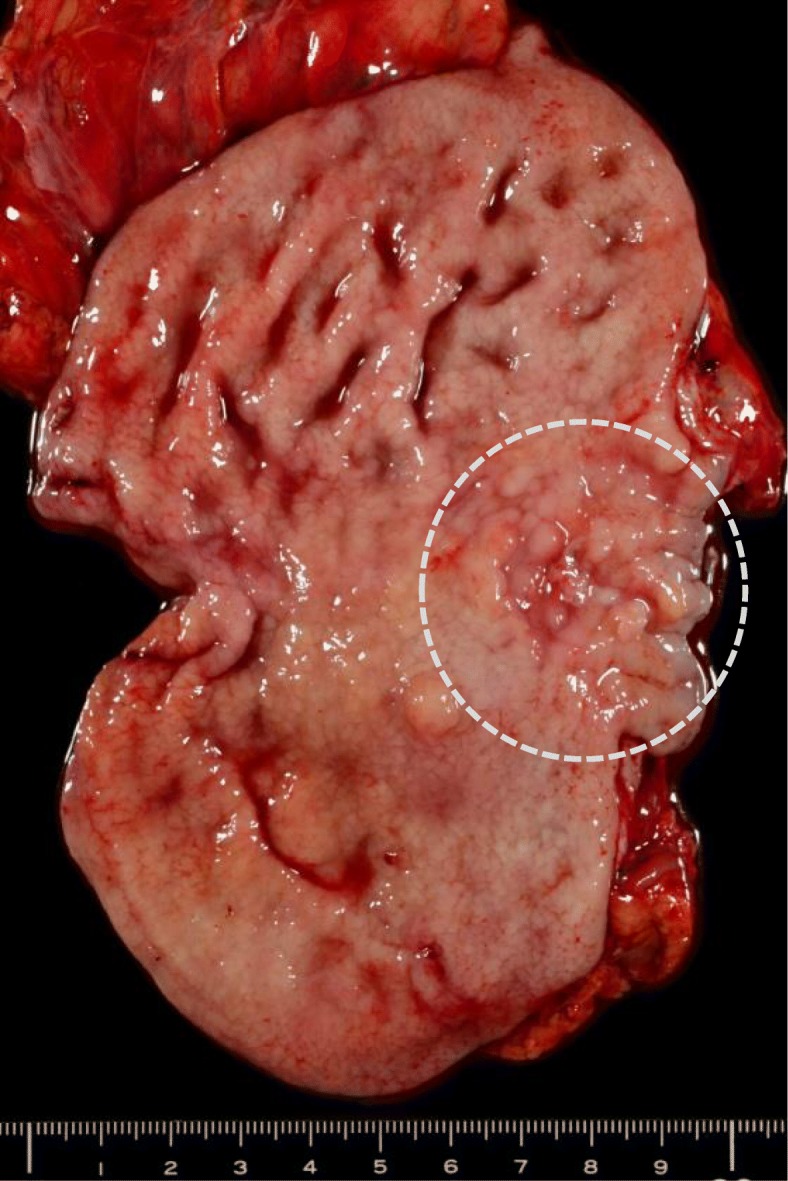
Macroscopic findings. Macroscopic findings of the resected a scar-like lesion in the lesser curvature of the remnant stomach

An 81-year-old man had been diagnosed with early gastric cancer and had undergone gastrectomy with Billroth I construction at 60 years of age. Currently, he underwent upper-gastrointestinal endoscopy for anemia that revealed an irregular lesion in the remnant stomach, for which he was referred to our hospital for further examination. Endoscopy and upper-gastrointestinal tract examination revealed type 3 advanced gastric cancer in the upper body of the stomach and slightly invading the esophagus. (Fig. 1). A biopsy specimen confirmed a poorly differentiated adenocarcinoma (Her-2 negative). An abdominal computed tomography (CT) scan showed the thickened gastric wall and two swollen PANs that were 70 mm and 30 mm in diameter, respectively (Fig. 2). We diagnosed the patient with unresectable RGC (Borrmann type 3, cT4a, cN1, cH0, cP0, cM1 (LYM), cStage IV according to the 7th UICC guidelines) and administered SOX chemotherapy. We expected that the tumor would be down staged after chemotherapy. S-1 (100 mg/body/day) was orally administered twice daily for the first 2 weeks of a 3-week course. Oxaliplatin was administered as an intravenous infusion of 150 mg/body/day on day 1 of each course. The patient completed three treatment courses without severe adverse effects, although he experienced mild but tolerable weakness and could continue treatment. Upper-gastrointestinal endoscopy after chemotherapy demonstrated that the gastric lesion had disappeared, and a gastric ulcer scar could be observed. Additionally, the abdominal CT revealed a reduction in the size of the PAN to 60% of the original mass. PET-CT was performed and there were no distant metastases. We thought an R0 resection was possible and considered an indication for conversion surgery. Hence, 36 days after the administration of the last dose of chemotherapy, we planned to perform radical surgery. Laparotomy findings showed no peritoneal metastasis, and peritoneal lavage cytology revealed no cancer cells in the abdominal cavity; we performed total remnant gastrectomy and D2 lymphadenectomy as well as PAN dissection with Roux-en-Y reconstruction. The time taken for surgery was 459 min, and the total blood loss was 340 mL. On macroscopic observation, ulcer scars were observed in the remnant stomach (Fig. 3). Microscopic examination revealed no tumor cells in the ulcer scar of the resected remnant stomach or in any of the lymph nodes including the PANs harvested from the surgical specimen. The therapeutic effect of SOX chemotherapy was grade 3, i.e., a complete response according to the Japanese guidelines on gastric cancer [1].

The patient’s postoperative course was uneventful, and the patient was discharged on postoperative day 24. Adjuvant chemotherapy with S-1 was performed in the outpatient setting, and the patient has remained disease-free for 1 year after surgery.

## Conclusions

RGC after distal gastrectomy accounts for 1–2% of cases among all gastric cancers in Japan [4, 5]. RGC is rare and is commonly detected at an advanced stage, resulting in low rates of curative resection (38–40%) and consequently, a poor prognosis [6, 7]. In advanced RGC, the incidence of lymph node metastasis is high because the lymphatic vessels have been transected during the initial surgery in the remnant stomach [8]. Specially as stage III and IV RGC has a poor prognosis [6, 7], combination treatment of chemotherapy and surgery is necessary for advanced RGC, but no standard treatment guidelines are available.

Conversion surgery is an option for stage IV gastric cancer when distant metastases are controlled with chemotherapy; however, the feasibility and efficacy of conversion surgery for gastric cancer remain unclear [9]. Among patients undergoing conversion surgery, the presence of one non-curative factor before surgery and performing R0 resection are predictors of a favorable OS [10]. Accordingly, conversion surgery may result in further long-term survival of selected patients [9].

The Japan Clinical Oncology Group (JCOG) 0405 trial was a phase II clinical study of preoperative S-1 plus cisplatin (CDDP) chemotherapy for gastric cancer with PANs and/or bulky lymph node enlargement but no other distant metastases [11]. Gastrectomy with extended lymph node dissection including PAN was performed after S-1/CDDP chemotherapy. A subsequent analysis showed a 5-year survival rate of 52.7% with good prognosis [11]. According to the updated Japanese guidelines on gastric cancer [1], S-1/CDDP chemotherapy is the first-line treatment and the first-level recommendation for HER 2-negative patients. This regimen is highly emetic and requires adequate hydration to prevent renal toxicity [2].

SOX is less toxic and more convenient as the first-line treatment for advanced gastric cancer (G-SOX study), because it does not require forced hydration, unlike CDDP, compared to CS [3]. And SOX is an effective and feasible therapy for elderly patients with advanced gastric cancer and demonstrated favorable efficacy and safety compared with CS [12]; however, the patients who will benefit from conversion surgery after SOX remain unclear.

In this patient, we chose the SOX regimen because the patient was elderly and because the SOX regimen would help protect renal and cardiac function. The recommended dose of chemotherapy was reduced because the patient was elderly and had previously undergone gastrectomy.

CT revealed that PANs had decreased in size. Hence, we diagnosed that the patient achieved a partial response, but as the pathological specimens showed no cancer cells, we believed that the patient had experienced a complete pathological response. This case could not be diagnosed as a complete clinical response because the lymph node metastasis had not completely disappeared on CT, even after three courses of chemotherapy; however, the resected specimen demonstrated a complete pathological response. Thus, a complete response may be difficult for gastric cancer despite chemotherapy. Therefore, conversion therapy may be required to perform resection considering the preoperative diagnosis of metastasis.

While there have been reports of a complete pathological response after chemotherapy with CDDP, a complete pathological response after SOX chemotherapy is rare. This is the first case of advanced gastric cancer that was treated with total remnant gastrectomy after SOX chemotherapy. Thus, preoperative SOX with surgery might be an effective treatment strategy for gastric cancer with PAN metastasis.

In conclusion, we encountered a patient with advanced RGC with giant PAN who showed a complete pathological response and favorable outcome after SOX chemotherapy. The findings of this case suggest that conversion therapy with SOX chemotherapy may be one of the treatments that may result in long-term survival of patients with unresectable gastric cancer.

## Abbreviations

CT: Computed tomography
PAN: Para-aortic lymph node metastasis
RGC: Remnant gastric cancer
SOX: S-1 and oxaliplatin
UICC: Union for International Cancer Control
